# Respiratory Syncytial Virus Versus Influenza in Hospitalized Adults During the Post-Pandemic Era: Clinical Characteristics, Similarities and Discrepancies

**DOI:** 10.3390/microorganisms14102379

**Published:** 2026-10-17

**Authors:** Eleni Papantoniou, Konstantinos Markakis, Iordanis Mimtsoudis, Eleni Georgianou, Dimitrios Pilalas, Sideris Nanoudis, Vasileios Nitsas, Vassilios Katsares, Theodoros Michailidis, Areti Tychala, Eleni Karlafti, Lemonia Skoura, Symeon Metallidis, Olga Tsachouridou

**Affiliations:** 1 1st Internal Medicine Department, AHEPA University Hospital, School of Medicine, Aristotle University of Thessaloniki, 54636 Thessaloniki, Greece; elenispap@yahoo.gr (E.P.); conmark@windowslive.com (K.M.); jordymim@hotmail.com (I.M.); elenigeorgi@hotmail.com (E.G.); pilalas_jim@hotmail.com (D.P.); sidnanoudis@yahoo.gr (S.N.); thgmichailidis@gmail.com (T.M.); linakarlafti@hotmail.com (E.K.); metallidissimeon@yahoo.gr (S.M.); 2 Department of Microbiology, “St’ Lukes” Hospital, 55236 Thessaloniki, Greece; vasilisnitsas@yahoo.gr (V.N.); vkatsare@gmail.com (V.K.); 3 Department of Microbiology, AHEPA University Hospital, School of Medicine, Aristotle University of Thessaloniki, 54124 Thessaloniki, Greece; aretich@gmail.com (A.T.); mollyskoura@gmail.com (L.S.)

**Keywords:** respiratory syncytial virus, influenza, hospitalized adults, multimorbidity, clinical outcomes, vaccination, post-pandemic era

## Abstract

Respiratory syncytial virus (RSV) has emerged as a major cause of hospitalization among adults in the post-pandemic era. We studied the epidemiological findings and the outcomes of patients hospitalized with influenza or RSV during the first post-pandemic respiratory virus season in Northern Greece. This multicenter retrospective study included adults hospitalized with RSV or influenza between November 2023 and May 2024 at two hospitals in Thessaloniki, Greece. Epidemiological data, clinical, laboratory, and radiological findings, vaccination status, and clinical outcomes were retrieved and compared. Twenty-seven patients with RSV and forty-eight with influenza were included. RSV patients were older (77.7 vs. 67.2 years, *p* < 0.01) with a higher prevalence of cardiovascular disease, chronic pulmonary disease, and malignancy, and had tachypnea more frequently at presentation (*p* = 0.024). No significant differences in the laboratory, radiological findings, and outcomes between the two groups were recorded. No patient was vaccinated against RSV, while 20.8% of the influenza patients had been previously vaccinated. RSV patients had a greater burden of comorbidities, whereas overall clinical outcomes did not significantly differ from those with influenza.

## 1. Introduction

Respiratory syncytial virus (RSV) is a major cause of acute respiratory tract infections (ARIs) and an increasingly recognized pathogen among adults, particularly those aged over 65 years and individuals with chronic comorbidities [1,2]. Globally, RSV is responsible for more than 4.5 million ARI cases and approximately 31,500 deaths annually. In Europe, an estimated 158,229 RSV-associated hospitalizations occur each year, predominantly among older adults [3].

Clinical manifestations range from mild upper respiratory tract symptoms to severe lower respiratory tract disease. Although most infections are self-limiting, approximately 12% of adults aged > 60 years with underlying conditions, including chronic cardiopulmonary disease, diabetes, renal impairment, or immunosuppression, develop severe illness requiring hospitalization [4]. Nearly one in five hospitalized RSV patients experience a severe outcome; 17% require intensive care unit (ICU) support and 4.8% require mechanical ventilation, while reported in-hospital mortality ranges from 6.6% to 12.8% [5].

Given its growing impact on older adults, RSV is increasingly recognized as an important cause of severe respiratory illness comparable to other major seasonal respiratory viruses, particularly influenza [6]. Contemporary data suggest that hospitalized RSV patients are typically older and carry a greater burden of underlying comorbidities, while experiencing clinical outcomes that are often similar to, or even worse than, those observed in influenza [7].

RSV traditionally followed a predictable seasonal pattern, with winter peaks in temperate regions. However, the COVID-19 pandemic and the implementation of non-pharmaceutical interventions substantially disrupted viral circulation, leading to reduced RSV activity followed by atypical post-pandemic resurgence and altered seasonality after the relaxation of restrictions [8,9,10,11]. At the same time, the prevention landscape has changed with the introduction of RSV vaccines for older adults and individuals at increased risk for severe disease. Three RSV vaccines have been approved since 2023 and have demonstrated more than 80% efficacy against severe RSV-associated lower respiratory tract disease in older adults [12,13,14,15], while preventive options against influenza have also expanded substantially in recent years [16].

In this evolving epidemiological landscape, a better understanding of the characteristics and outcomes of RSV infection in adults is increasingly important. Therefore, this study aimed to describe the epidemiological, clinical, and laboratory features of adults hospitalized with RSV during the first post-pandemic respiratory season and to compare their characteristics, disease severity, and outcomes with those of patients hospitalized with influenza. By providing real-world data from Northern Greece during the initial rollout of RSV vaccination, this analysis seeks to further define the burden of RSV-associated hospitalization in adults and support preventive strategies targeting high-risk populations.

## 2. Materials and Methods

### 2.1. Study Population

This was a two-center retrospective study including adults hospitalized with RSV- or influenza-associated ARIs between 1 November 2023 and 1 May 2024. The study was conducted at AHEPA University Hospital and St. Luke’s Hospital in Thessaloniki, Northern Greece. Eligible participants were adults aged ≥ 18 years, admitted and subsequently hospitalized with symptoms of upper or lower respiratory tract infection and/or systemic manifestations such as fever, rigors, or chills, who were identified in the routine screening for influenza and RSV and had a positive nasal swab confirmed by real-time polymerase chain reaction (RT-PCR) within 48 h of hospital admission. The RT-PCR assays applied were either the NeuMoDx™ Flu A-B/RSV/SARS-CoV-2 Vantage Assay on the NeuMoDx™ 96 Molecular System (QIAGEN, Hilden, Germany) or the Xpert^®^ Xpress CoV-2/Flu/RSV plus assay on the GeneXpert^®^ System (Cepheid, Sunnyvale, CA, USA). The primary outcomes were to:

(a) Record the epidemiological, clinical, laboratory, and radiological findings of the patients diagnosed with influenza or RSV infection
(b) Find differences between the two groups
(c) Record the clinical outcomes (non-invasive and invasive ventilation, ICU length of stay, total length of stay, death) of the eligible patients

The secondary outcome was to

(a) explore associations between clinical, laboratory, epidemiological, and radiological characteristics and clinical outcomes using univariable statistical and correlation analyses.

Epidemiological, clinical, and laboratory data were collected, including age, sex, medical comorbidities, concurrent medications, clinical signs, and symptoms (fever, cough, dyspnea, tachypnea, and desaturation), and radiological findings on chest radiography or computed tomography (CT). Information regarding the need for high-flow oxygen therapy, non-invasive (NIV) or invasive mechanical ventilation (IMV) was also recorded. Laboratory parameters included leukocyte count, neutrophil-to-lymphocyte ratio, C-reactive protein (CRP), procalcitonin (PCT), ferritin, D-dimers, and platelet count. Hospital length of stay, ICU length of stay, duration of NIV support, and clinical outcome were additionally assessed. Vaccination status for RSV, influenza, and SARS-CoV-2, as well as a history of viral respiratory infection during the preceding six months, were also recorded. Prior immunization was recorded based on the electronic prescribing system records.

### 2.2. Statistical Analysis

Data distribution was assessed using the Kolmogorov–Smirnov test. Normally distributed variables are presented as mean ± standard deviation (SD), whereas non-normally distributed variables are presented as median and interquartile range (IQR). Comparisons between groups were performed using the independent-samples *t*-test for parametric variables and the Mann–Whitney U test for non-parametric variables.

Pearson’s or Spearman’s correlation analyses were performed, as appropriate, to evaluate associations between epidemiological, clinical, and laboratory characteristics and clinical outcomes, including hospital length of stay, need for high-flow oxygen therapy, need for NIV or IMV support, and mortality. Categorical variables were compared using the χ^2^ test or Fisher’s exact test. A two-sided *p*-value < 0.05 was considered statistically significant. Adjustment for multiple comparisons was performed with the Benjamini–Hochberg false discovery rate (FDR) analysis for the prespecified exploratory comparisons, and an adjusted *p*-value (*q*-value) < 0.05 was considered significant. Binary logistic regression was applied for age-adjusted comparisons. Moreover, binary logistic regression analyses for every outcome (death, NIV, IMV) were performed. All statistical analyses were performed using SPSS Statistics version 26 (SPSS Inc., Chicago, IL, USA).

## 3. Results

**Figure 1:**
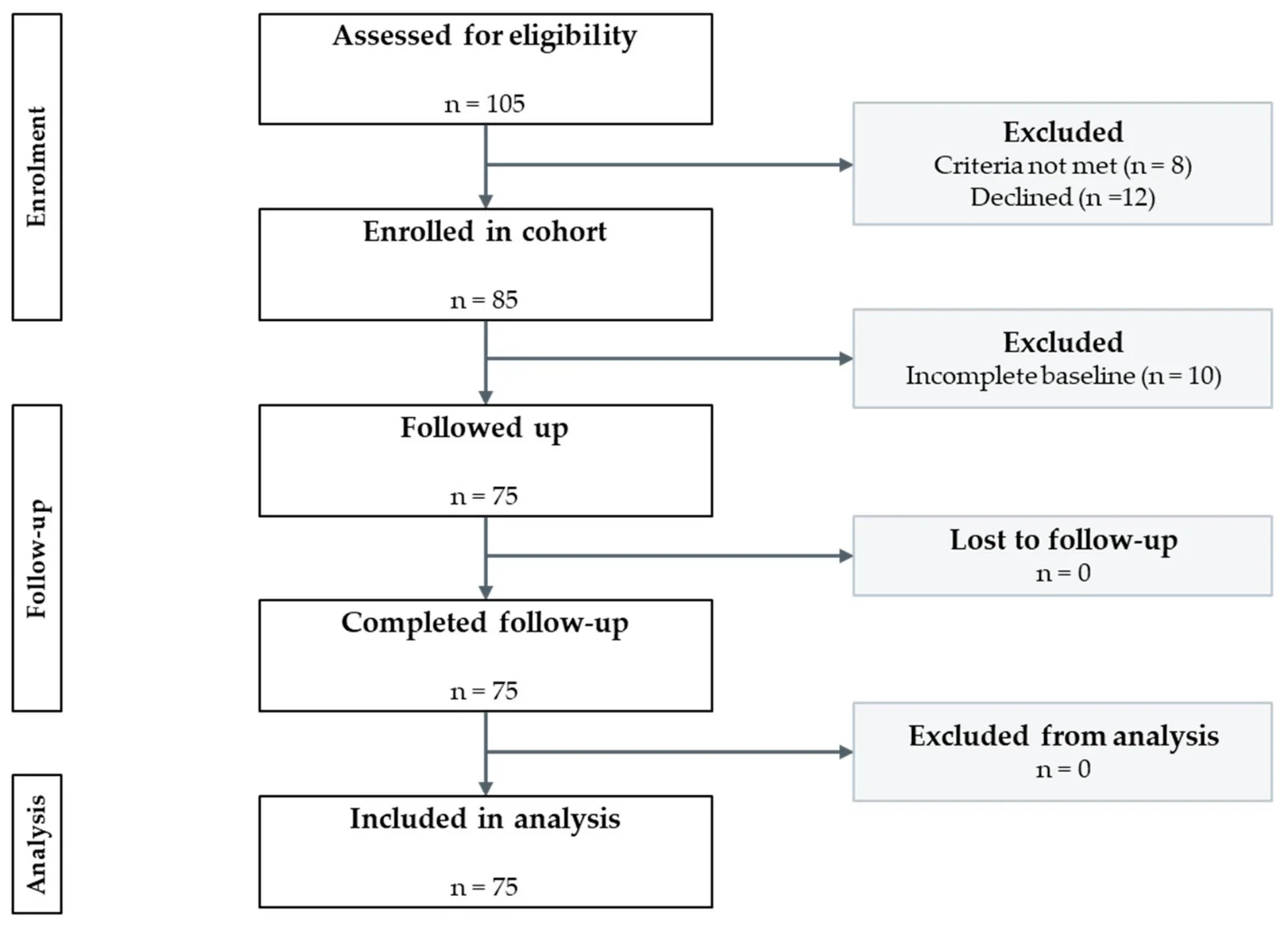
Participant flow through the study. Based on the template from: von Elm E, Altman DG, Egger M et al. “The Strengthening the Reporting of Observational Studies in Epidemiology (STROBE) statement” [17].

A total of 75 hospitalized adults with laboratory-confirmed RSV or influenza infection were included in the study, including 27 RSV and 48 influenza cases (Figure 1).

**Figure 2:**
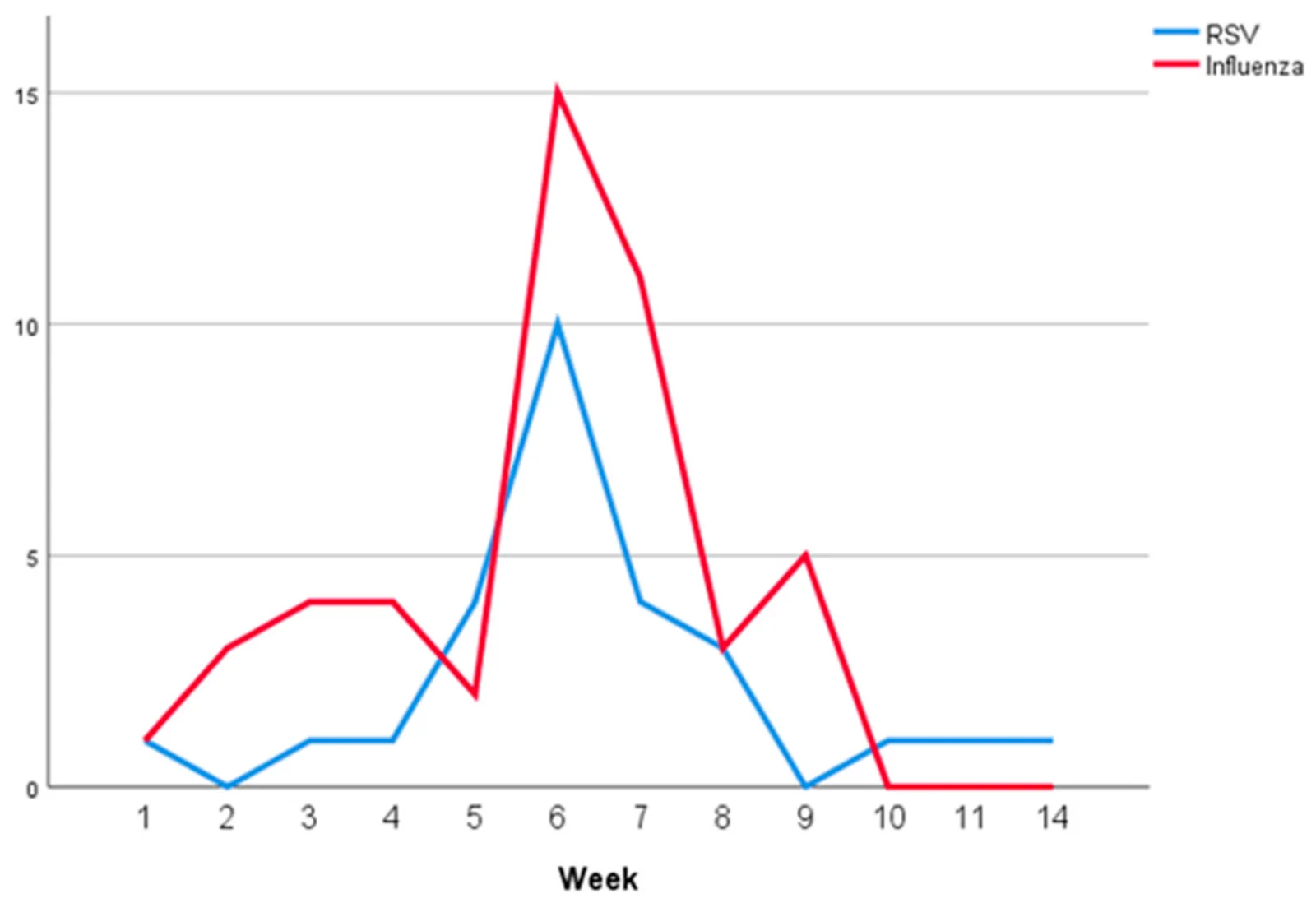
Weekly distribution of RSV and influenza hospitalizations during the study period.

The weekly distribution of RSV and influenza hospitalizations is presented in Figure 2:

### 3.1. RSV Infection

During the study period, 27 patients were hospitalized with RSV infection, including 16 men (59.3%) and 11 women (40.7%). The mean age was 77.7 ± 9.9 years. Dyspnea was the most common presenting symptom, observed in 17 patients (63%), followed by fever and tachypnea, each recorded in 15 patients (55.6%). Cough was reported in 11 patients (40.7%), while desaturation at admission, defined as oxygen saturation < 90% on room air, was reported in 17 patients (63%).

The median CRP level was 3.86 mg/dL (IQR 8.86), median PCT was 0.17 ng/mL (IQR 0.325), median ferritin was 182.5 ng/mL (IQR 234), and median neutrophil-to-lymphocyte ratio was 6.7 (IQR 7.6). The median platelet count was 196,000/μL (IQR 108,000), while median D-dimer levels were 440.5 ng/mL (IQR 579). Corticosteroids were administered to 16 patients (59.3%), and none of the patients had received RSV vaccination. Pulmonary infiltrates were identified on chest radiography or CT in 16 patients (59.3%).

Five patients (18.5%) required NIV, for a median duration of 5 days (IQR 6.5). Four patients (14.8%) required IMV and were admitted to the ICU, where the median length of stay was 11 (IQR 7) days. Overall, six patients died during hospitalization, corresponding to an in-hospital mortality rate of 22.2%. The median duration of hospitalization was 9 days (IQR 10).

Among patients with RSV infection, mortality was significantly associated with arterial hypertension (*p* = 0.016), the need for NIV support (*p* < 0.01), and the requirement for IMV (*p* = 0.025). Nonetheless, after FDR correction analysis, the statistical significance of these parameters was lost (arterial hypertension q = 0.224; NIV q = 0.112; IMV q = 0.233). No significant associations were observed between mortality and inflammatory biomarkers, presenting clinical features, other evaluated comorbidities, corticosteroid administration, or previous vaccination status.

The need for NIV was significantly associated with arterial hypertension (*p* = 0.041) and tachypnea at presentation (*p* = 0.047). However, after adjustment for multiple comparisons, neither of those parameters retained statistical significance (q = 0.486 for both).

As expected, NIV duration was strongly correlated with total hospital length of stay (*r* = 0.914, *p* = 0.03). However, the corresponding Spearman rank correlation was not statistically significant (ρ = 0.872, *p* = 0.054). Taking into account the small sample size with only five patients treated with NIV, the finding should not be interpreted as a statistically significant association but rather considered exploratory.

### 3.2. Influenza Infection

A total of 48 patients were hospitalized with influenza infection during the study period, with an equal distribution between men and women (24 patients each). The mean age was 67.2 ± 16.4 years. All infections were caused by Type A influenza, whereas Type B was not detected in any of the patients. Fever was the most common presenting symptom, recorded in 28 patients (58.3%), followed by dyspnea in 22 (45.8%), cough in 15 (31.3%), and tachypnea in 14 (29.2%). Desaturation at admission was observed in 25 patients (52.1%).

In our cohort, the patients with influenza had a median CRP level of 8.1 mg/dL (IQR 13.5), PCT of 0.12 ng/mL (IQR 0.96), ferritin of 419 ng/mL (IQR 681.25), and median D-dimer levels of 444 ng/mL (IQR 651). Moreover, the median neutrophil-to-lymphocyte ratio was 6.8 (IQR 5.65), while the median platelet count was 202,500/μL (IQR 87,500). Pulmonary infiltrates on chest radiography or CT were identified in 22 patients (45.8%).

Regarding prior vaccination, one patient (2.1%) had received the standard quadrivalent (QIV), four (8.3%) the adjuvanted quadrivalent (aQIV), and five (10.4%) the high-dose quadrivalent influenza vaccine (QIV-HD). All hospitalized patients received oseltamivir as standard of care, while corticosteroids were administered to 21 patients (43.8%) during hospitalization.

Six patients (12.5%) required NIV support for a median duration of 4 days (IQR 9), while five patients (10.4%) required IMV. Among patients admitted to the ICU, the median ICU length of stay was 5 (IQR 2) days, while the median duration of hospitalization was 7 days (IQR 5.75). Overall, 11 patients died during hospitalization, corresponding to an in-hospital mortality rate of 22.9%.

Among patients hospitalized with influenza, several laboratory inflammatory markers were significantly associated with mortality in the univariable analysis. Non-survivors had higher CRP (*U* = 327.5, *p* < 0.01), PCT (*U* = 54.5, *p* < 0.01), ferritin (*U* = 184, *p* = 0.022), and D-dimer levels (*U* = 302, *p* = 0.016) compared with survivors. A history of malignancy was also associated with an increased risk of death (*p* = 0.01). Moreover, NIV and IMV during hospitalization were also associated with an increased risk of death (*p* = 0.001 and <0.001, respectively). Nonetheless, after adjustment for multiple comparisons (FDR analysis), IMV (q = 0.01), NIV (q = 0.02), CRP (q = 0.02), and PCT (q = 0.05) remained statistically significantly associated with death, while the remaining variables did not retain their statistical significance (malignancy q = 0.07; D-dimers q = 0.09; ferritin q = 0.1).

The need for NIV was associated with corticosteroid administration during hospitalization (*p* < 0.01) and a history of chronic respiratory disease, including COPD or asthma (*p* = 0.05). However, after performing FDR analysis for multiple comparisons, the nominal association with corticosteroids and chronic respiratory disease did not remain statistically significant (q = 0.120 for corticosteroids; q = 0.75 for chronic respiratory diseases). Patients requiring NIV were more likely to undergo IMV (*p* = 0.01) and had a worse prognosis than those who did not require NIV support (*p* < 0.01). As expected, NIV duration correlated positively with total hospital length of stay (*r* = 0.828, *p* = 0.042).

Among patients’ medical comorbidities, malignancy (*U* = 122, *p* = 0.014) was associated with longer hospitalization. Among laboratory parameters, only D-dimer levels correlated significantly with hospital length of stay (*r* = 0.345, *p* = 0.016). After adjustment for multiple comparisons, D-dimer levels, history of malignancy, and duration of NIV did not retain their statistical significance (q = 0.0.27; q = 0.27; q = 0.48, respectively).

### 3.3. RSV vs. Influenza Infection

The female-to-male ratio was similar between the two groups. However, patients hospitalized with RSV were significantly older than those admitted with influenza (77.7 vs. 67.2 years, *p* < 0.01) and exhibited significantly higher rates of several chronic comorbidities, including hyperlipidemia, coronary heart disease, heart failure, atrial fibrillation, chronic pulmonary disease, and malignancy (Table 1). Nonetheless, after adjustment for age, the different prevalence of hyperlipidemia between patients with RSV and influenza infection was no longer statistically significant (adjusted OR 2.71, 95% CI 0.88–8.29; *p* = 0.08). Similarly, coronary heart disease and heart failure prevalence between the two groups were no longer significantly different after age adjustment (coronary heart disease: adjusted OR 2.74, 95% CI 0.8–9.41; *p* = 0.11; heart failure: adjusted OR 2.8, 95% CI 0.8–9.7; *p* = 0.11). The prevalence of atrial fibrillation, chronic pulmonary disease, and malignancy remained significantly different between the two groups after adjusted for age (atrial fibrillation: OR 9.03, 95% CI 2.17–37.5; *p* = 0.002; chronic pulmonary disease: OR 3.76, 95% CI 1.23–11.44; *p* = 0.02; malignancy: OR 5.22, 95% CI 1.18–23.04; *p* = 0.029). Notably, patients with RSV had received an influenza vaccine in the last vaccination period significantly more often than influenza patients (*p* = 0.03). Nonetheless, after adjustment for age, the difference did not retain statistical significance.

Among presenting clinical features, tachypnea was more common in patients with RSV than in those with influenza (55.6% vs. 29.2%, *p* = 0.024), whereas with respect to the frequency of fever, cough, dyspnea, and desaturation, no significant difference between the two groups was observed. Likewise, the laboratory findings were similar between the two groups, and radiological findings were recorded with similar frequency. Nonetheless, as explained later, the lack of any significant difference between the two groups should not be regarded as evidence of equality due to the small sample size.

The proportion of patients receiving corticosteroids during hospitalization was similar in both groups. Interestingly, the interval between a previous SARS-CoV-2 infection and the current hospitalization was shorter among patients with RSV than among those with influenza (12 vs. 18 months, *p* = 0.049). Among patients admitted to the ICU, those with RSV had a significantly longer ICU stay than those with influenza (11 [IQR 7] vs. 5 days [IQR 2], *p* = 0.017, *U* = 2.5, *z* = −2.31, 95% *CI* [0.00, 10.00]). However, overall only six patients with RSV infection and five with influenza were admitted to the ICU, making it inappropriate to draw any conclusions regarding differences in infection severity between the two groups. Given the difference in age between the groups, an exploratory linear regression model was additionally performed with ICU length of stay as the dependent variable and infection type and age as independent variables. After adjustment for age, RSV infection remained associated with a longer ICU stay (adjusted mean difference, 6.81 days; 95% CI, 1.10–12.51; *p* = 0.025). Given the very small number of patients requiring ICU admission (n = 11), this adjusted analysis should be considered exploratory, and the findings interpreted accordingly.

Regarding in-hospital mortality, total hospital length of stay, or the need for NIV or IMV, no significant difference between the two groups was observed. As mentioned before and explained later, due to the study design, these findings should not be interpreted as evidence of equivalence.

### 3.4. Exploratory Multivariable Regression Analysis

To account for potential confounding by baseline differences between the groups, multivariable logistic regression analyses were performed for mortality, NIV, and IMV. Given the small sample size and limited number of outcome events, parsimonious models were used, including infection group (RSV vs. influenza), age, sex, and overall comorbidity burden, defined as the number of the 11 prespecified chronic comorbidities assessed in the study. After adjustment, RSV infection was not significantly associated with risk of death, NIV, or IMV (death: adjusted OR 2.105, 95% CI 0.534–8.305; *p* = 0.288; NIV: adjusted OR 1.058, 95% CI 0.229–4.894; *p* = 0.943; IMV: adjusted OR 0.625, 95% CI 0.107–3.655; *p* = 0.6). Similarly, none of the other variables were significantly associated with the evaluated outcomes (Table 2). The adjusted analyses were considered exploratory given the limited number of outcome events.

## 4. Discussion

In this study, we recorded the clinical characteristics and outcomes of adults hospitalized with RSV and influenza in the post-pandemic era, highlighting both shared and distinct clinical patterns. Patients with RSV were significantly older and exhibited a greater burden of comorbidities, including coronary heart disease, heart failure, atrial fibrillation, chronic pulmonary disease, and malignancy. RSV infection was also associated with a higher frequency of tachypnea at presentation. Despite these differences, laboratory and radiological findings were comparable between the two groups. Moreover, in-hospital mortality, total length of stay, and the need for NIV or IMV between influenza and RSV patients were not statistically different. Nonetheless, given the small sample size of our cohort, the study may have been underpowered to detect clinically relevant differences between influenza and RSV patients. Therefore, the absence of statistically significant differences should not be interpreted as evidence of equivalence, and these findings should be considered exploratory and hypothesis-generating, requiring confirmation in larger cohorts. Similarly, the recorded longer ICU stay in RSV patients cannot be used to infer a significant difference in infection severity between the RSV and the influenza group, as the sample size was small (6 RSV and 5 influenza patients). Thus, it should be interpreted only as an exploratory finding.

All influenza infections in our cohort were caused by Type A influenza. The absence of any influenza Type B infections is in agreement with the epidemiological data of the National Organism of Public Health (EODY), which, for the epidemiological period of 2023–2024, reported a predominance of severe influenza infections caused by Type A (92%), whereas Type B accounted for only 8% of the severe infections in the Greek region. Moreover, the great majority of Type B infections in the Greek region were recorded after February 2024, whereas in our cohort, no cases of influenza were recorded after February 2024, which can explain the lack of any Type B infections. The older age and greater burden of comorbidities identified among RSV patients in our cohort are consistent with numerous reports from diverse healthcare settings. Adults hospitalized with RSV tend to be older than those admitted with influenza (65.5 vs. 58 years), while 95.5% of individuals aged ≥ 60 years exhibit at least one underlying medical condition [18]. Although cardiovascular and chronic pulmonary diseases predominate, affecting more than half of patients, nearly 30% present with diabetes, chronic kidney disease, neurologic disorders, or immunocompromising conditions, highlighting the extensive multimorbidity associated with RSV hospitalization [19]. Heart failure is reported in 35.3–37.3% and COPD in 29.8–47.6% of hospitalized RSV patients, consistently exceeding the corresponding rates observed in influenza, whereas asthma and solid malignancies are also more common among RSV hospitalized adults [20,21]. In our study, heart failure and chronic pulmonary disease were approximately three times more prevalent among RSV than influenza patients. However, our cohort also demonstrated a broader cardiovascular profile, encompassing significantly higher rates of coronary heart disease and atrial fibrillation. Nonetheless, after adjustment for age, only atrial fibrillation, chronic pulmonary disease, and malignancy were still significantly more prevalent among RSV patients, while the other comorbidities no longer had a different prevalence between the groups. Previous studies have also recorded preexisting CVD, chronic pulmonary diseases, and malignancy as common comorbidities in patients hospitalized with RSV, which can affect the outcomes [22,23]. Conversely, recent studies highlight the impact of RSV on patients in regard to cardiovascular events [24]. Notably, CVD is reported in 56.4% of hospitalized adults, and acute cardiac events occur in 22.4% of cases, both of which were associated with significantly increased risks of ICU admission and in-hospital mortality [25].

Despite these important differences in baseline characteristics, the clinical presentation of RSV and influenza infections exhibited substantial overlap among our patients. Tachypnea was the only symptom that occurred significantly more frequently among RSV patients, whereas the prevalence of fever, cough, dyspnea, and desaturation did not differ between the two groups. Similar observations have been previously reported, although modest differences in symptom patterns have been described. Lower rates of fever but a significantly higher frequency of dyspnea and wheezing have been reported among RSV patients, affecting 33.8% and 29.7% of cases, respectively, compared with 10.9% and 5.4% among patients hospitalized with influenza [18]. Furthermore, a higher prevalence of productive cough has been documented among RSV patients, occurring in 62.3% of cases compared with 50.7% among influenza patients, while fever was more frequently observed in influenza, affecting 64.8% and 49.4% of patients, respectively [26]. Overall, RSV was predominantly characterized by cough, fever, dyspnea, and respiratory distress, whereas influenza was more commonly associated with upper respiratory tract symptoms, including headache, nasal congestion, and sore throat [27]. Nevertheless, these differences were not sufficiently consistent across the literature to define a distinct clinical phenotype for either infection, as no clear clinical characteristics capable of reliably distinguishing RSV from influenza among hospitalized adults have emerged [28].

Laboratory and radiological findings did not particularly differentiate RSV from influenza in our cohort, as CRP, PCT, ferritin, and D-dimer levels, as well as the frequency of pulmonary infiltrates, were comparable between the two groups, notwithstanding the small sample size. Supporting evidence comes from previous studies, with no significant differences in CRP levels despite higher white blood cell and lymphocyte counts among RSV patients [29]. In contrast, other investigators reported a greater prevalence of laboratory abnormalities in RSV, with elevated CRP present in 70.0% of RSV cases compared with 63.7% of influenza cases and leukocytosis in 34.2% versus 26.2% of patients, while pulmonary infiltrates have been identified in 41% of RSV patients compared with 29% and 24% of those with influenza A and influenza B, respectively [22,30]. These heterogeneous results suggest that laboratory and radiological abnormalities are more likely to reflect methodological differences between cohorts, including age-stratified analyses, variations in age distribution, comorbidity burden, and disease severity, rather than reproducible disease-specific characteristics.

When major clinical outcomes were examined, no statistically significant differences emerged between RSV and influenza patients regarding mortality, ventilatory support requirements, or overall hospital length of stay; notwithstanding that, due to the small sample size and the limited outcome events, residual confounding cannot be excluded. Likewise, in the exploratory multivariable regression analysis we conducted, age, sex, infection type, and comorbidity burden were not associated with the outcomes. Consistent with our findings, several studies have reported comparable in-hospital outcomes between RSV and influenza, despite a higher baseline risk profile among RSV patients. In a cohort of 639 hospitalized adults, similar 30-day mortality rates were reported between RSV and influenza, as well as comparable rates of ICU admission, vasopressor support, and mechanical ventilation, although the median length of stay was longer in the RSV group [31]. In a multicenter analysis of three major respiratory viruses, no significant differences were observed in ICU admission or in-hospital mortality between RSV and influenza among adults [32]. In contrast, other studies indicated a higher likelihood of mechanical ventilation among RSV patients compared to those with influenza, although in-hospital mortality remained comparable at approximately 5% in both groups [33]. Moreover, one large population-based study reported more than fourfold higher adjusted odds of 28-day mortality and more than twofold higher adjusted odds of ICU admission among RSV patients compared with those hospitalized for influenza [34]. Importantly, analyses accounting for differences in baseline characteristics have suggested that much of the apparent excess severity associated with RSV may be attributed to the older age and greater comorbidity burden of affected patients, as demonstrated in a propensity-weighted analysis where differences in mortality were no longer significant, and the association between RSV infection and severe outcomes was substantially attenuated after balancing for baseline differences between the two groups [35]. Interestingly, in our cohort, the in-hospital mortality was higher than in other recent studies [36,37,38]. Differences in the epidemiological characteristics, age, as well as in the incidence of preexisting medical conditions between the examined populations could be the reason for this discrepancy. In particular, in the study of Celante et al., a mortality of 6.6% was reported, significantly lower than in our cohort [36]. Nonetheless, the patients in our group were older, with a mean age of 77.7 and median age of 80 (IQR 17) vs. 75 (IQR 22) years in the study of Celante. Importantly, certain medical preconditions, specifically COPD and a malignancy, were much more common in our cohort with RSV patients (48.1% vs. 28.6% and 29.6% vs. 10.1%, respectively). Likewise, in the Hovind cohort, the patients with active malignancy or diabetes mellitus were relatively fewer than in our population [37]. Similarly, in a retrospective, real-world analysis with over 170,000 patients with documented RSV infection, which was the largest in size included in the meta-analysis by Yayan and, as such, is of increased importance, while the median age was similar to our study (79 years old), the male/female ratio was significantly different, with women being the majority of documented cases (64.8) [38,39]. In addition, the patients under chemotherapy represented only 5.1% of the included patients, which differs from the prevalence of malignancy in our cohort. Taken together, these differences could provide a possible explanation for the high mortality in our cohort. Despite similar rates of NIV, IMV, and mortality between the two groups, RSV patients remained in the ICU for more than twice as long as influenza patients (10 vs. 4.6 days). In a cohort of more than 13,000 RSV-related hospitalizations, ICU stays exceeded 10 days among mechanically ventilated RSV patients, while a large population-based analysis demonstrated a 77% longer ICU stay among RSV hospitalizations compared with influenza [34,40]. However, though the ICU length of stay of patients with RSV was longer than that of influenza patients in our cohort, that finding should be interpreted only as exploratory, as the total number of patients admitted was very low (6 with RSV vs. 5 with influenza), which limits the significance of that observation.

From a public health perspective, these observations highlight the importance of effective preventive strategies in high-risk populations. During the study period, none of the patients in our cohort had received RSV vaccination, while influenza vaccination coverage was also suboptimal, at approximately 20%. Unlike influenza, for which the benefits of seasonal vaccination in preventing severe disease and hospitalization have long been established [41], RSV vaccines have only recently been introduced into clinical practice, and their population-level impact is still emerging. In particular, the Greek national vaccination committee, in its updated recommendation published in September 2024, recommended the inclusion of the RSV vaccine in the National Immunization Program for Adults. As a result, the RSV vaccine was incorporated into the National Immunization Program for Adults for the year 2025. Their development has been driven by the need to overcome immunosenescence through vaccine platforms capable of eliciting robust immune responses in older adults, who represent the population at greatest risk for severe RSV disease [42]. Recent real-world evidence is encouraging, demonstrating approximately 60% effectiveness against RSV-associated hospitalization and nearly 70% effectiveness against ICU admission or death among adults aged ≥ 60 years [43]. As clinical experience accumulates, future studies will elucidate whether the promising results observed to date translate into sustained protection and meaningful reductions in RSV-associated severe clinical outcomes.

This study has several important strengths that merit consideration. It included consecutive adults hospitalized with RSV or influenza across two different healthcare settings, comprising one public tertiary-care hospital and one private hospital serving Northern Greece. Restricting inclusion to patients with laboratory-confirmed infection within 48 h of admission ensured that hospitalizations were attributable to the viral infection under investigation rather than incidental viral detection, while the systematic collection of vaccination status and recent SARS-CoV-2 infection further strengthened the clinical characterization of the study population. Importantly, this analysis contributes contemporary post-pandemic data on adult RSV infection from Greece, a setting for which published evidence remains scarce.

The findings of this study should be interpreted in light of certain limitations. Its retrospective design is inherently susceptible to incomplete documentation and residual confounding, while differences in testing practices between the two participating hospitals may also have introduced selection or information bias. A further limitation of our study is that the ICU length-of-stay analysis is, as mentioned before, based on extremely small subgroups. Given the limited number of participants and outcome events, particularly in the RSV subgroup, the analyses should be considered exploratory and hypothesis-generating rather than predictive. Hence, the findings should be interpreted with caution. In addition, ICU admission depends on disease severity, comorbidity, and bed availability, and it may introduce collider or selection bias;

In addition, detailed information regarding specific immunocompromising conditions, such as active chemotherapy or solid organ transplantation, was not available, precluding assessment of their impact on clinical outcomes. Moreover, the absence of post-discharge follow-up precluded the assessment of longer-term outcomes, while influenza A and B infections were analyzed together, preventing subtype-specific comparisons. Importantly, vaccine effectiveness was not among the outcomes of the current study. Specifically, none of the RSV patients had been vaccinated, while approximately 20% of influenza patients had received influenza vaccination. Due to the study design and the small sample, no conclusions on vaccine effectiveness and impact on prevention can be made.

## 5. Conclusions

To our knowledge, this is the first study assessing patients hospitalized with RSV or influenza infection in the Greek area in the immediate post-pandemic time period before the novel vaccines were widely available. It supports the growing recognition that RSV and influenza are associated with comparable overall severity among hospitalized adults, despite distinct clinical characteristics. RSV predominantly affected older adults with a greater burden of cardiovascular and pulmonary comorbidities. In general, no greater differences in the primary clinical outcomes—death, total hospital stay, NIV, and IMV need- were recorded. The recorded longer ICU stay in RSV patients should be considered an exploratory and hypothesis-generating finding only, as the number of patients admitted to the ICU was limited (only five patients with influenza and six with RSV). Overall, though largely exploratory, these findings reinforce the need to recognize RSV as a cause of hospitalization in older adults and support ongoing efforts to optimize early diagnosis and clinical management in high-risk populations.

## Figures and Tables

**Table 1:** Comparison of demographic, clinical, laboratory, and outcome characteristics of RSV and influenza hospitalized adults.

	RSV (N/%)	Influenza (N/%)	*p*-Value	Test	Age-Adjusted Significance
**Number (N)**	27	48			
**Age (years)**	77.7 (±9.9)	67.2 (±16.4)	**<0.01**	*t*-test	
**Sex (male/%)**	16 (59.3%)	24 (50%)	0.44	Chi-square	
**Comorbidities**					
Arterial hypertension	14 (51.9%)	19 (39.6%)	0.3	Chi-square	0.87
Diabetes mellitus	7 (25.9%)	13 (27.1%)	0.9	Chi-square	0.77
Hyperlipidemia	14 (51.9%)	10 (20.8%)	**<0.01**	Chi-square	0.08
Coronary heart disease	11 (40.7%)	7 (14.6%)	**0.011**	Chi-square	0.11
Atrial fibrillation	12 (44.4%)	3 (6.3%)	**<0.01**	Chi-square	**0.002**
Heart failure	10 (37%)	6 (12.5%)	**0.013**	Chi-square	0.11
Chronic pulmonary disease	13 (48.1%)	8 (16.7%)	**<0.01**	Chi-square	**0.02**
Chronic kidney disease	3 (11.1%)	1 (2.1%)	0.13	Fisher-exact	0.24
Stroke	1 (3.7%)	1 (2.1%)	1	Fisher-exact	0.84
Malignancy	8 (29.6%)	3 (6.3%)	**0.014**	Fisher-exact	**0.029**
Autoimmune disease	2 (7.4%)	4 (8.3%)	1	Fisher-exact	0.99
**Prior vaccination status**					
Previous influenza vaccine	12 (44.4%)	10 (20.8%)	**0.03**	Chi-square	
Time since last COVID-19 vaccine (months)	24 (IQR 3.5)	23 (IQR 5.5)	0.22	Mann–Whitney-U	
Time since last COVID-19 infection (months)	12 (IQR 14)	18 (IQR 9)	**0.049**	Mann–Whitney-U	
Corticosteroid administration	16 (59.3%)	21 (43.8%)	0.2	Chi-square	
**Clinical signs and symptoms**					
Fever	15 (55.6%)	28 (58.3%)	0.82	Chi-square	
Cough	11 (40.7%)	15 (31.3%)	0.41	Chi-square	
Dyspnea	17 (63%)	22 (45.8%)	0.15	Chi-square	
Tachypnea	15 (55.6%)	14 (29.2%)	**0.024**	Chi-square	
Desaturation	17 (63%)	25 (52.1%)	0.36	Chi-square	
**Radiological findings (CT)**	16 (59.3%)	22 (45.8%)	0.72	Chi-square	
**Laboratory findings**					
Neutrophil/Lymphocyte ratio	6.7 (IQR 7.6)	6.8 (IQR 5.65)	0.56	Mann–Whitney U	
Platelets (10^3^/μL)	196 (IQR 108)	202.5 (IQR 87.5)	0.65	Mann–Whitney U	
CRP (mg/dl)	3.9 (IQR 8.86)	8.1 (IQR 13.5)	0.23	Mann–Whitney U	
PCT (ng/ml)	0.17 (IQR 0.33)	0.12 (IQR 0.96)	0.55	Mann–Whitney U	
Ferritin (ng/ml)	182.5 (IQR 234)	419 (IQR 681.25)	0.08	Mann–Whitney U	
D-Dimers (ng/ml)	440.5 (IQR 579.3)	444 (IQR 651)	0.83	Mann–Whitney U	
**Increased ventilation assistance**					
NIV	5 (18.5%)	6 (12.5%)	0.51	Fisher-exact	
NIV-duration (days)	5 (IQR 6.5)	4 (IQR 9)	0.57	Mann–Whitney U	
Invasive mechanical ventilation	4 (14.8%)	5 (10.4%)	0.71	Fisher-exact	
**ICU length of stay (days)**	11 (IQR 7)	5 (IQR 2)	**0.017 (CI 0.00–10.00)**	Mann–Whitney U	**0.025 (CI 1.10–12.51)**
**Total duration of hospitalization (days)**	9 (IQR 10)	7 (IQR 5.75)	0.1	Mann–Whitney U	
**Death**	6 (22.2%)	11 (22.9%)	0.95	Chi-square	

CRP: C-reactive protein; CT: computer tomography; PCT: procalcitonin; NIV: non-invasive ventilation; IMV: invasive mechanical ventilation; ICU: intensive care unit. Statistically significant *p*-values are shown in bold.

**Table 2:** Multivariable logistic regression analysis with outcomes of death, NIV, and IMV. Multivariable logistic regression models included infection group, age, sex, and comorbidity burden. RSV was compared with influenza; female sex was the reference category, and comorbidity burden was defined as the number of the 11 prespecified chronic comorbidities.

Predictor	Mortality aOR (95% CI)	*p*	NIV aOR (95% CI)	*p*	IMV aOR (95% CI)	*p*
RSV vs. influenza	2.105 (0.534–8.305)	0.288	1.058 (0.229–4.894)	0.943	0.625 (0.107–3.655)	0.6
Age, per year	1.004 (0.958–1.052)	0.872	0.996 (0.941–1.054)	0.885	0.919 (0.842–1.003)	0.057
Male sex vs. female	0.734 (0.238–2.262)	0.590	0.971 (0.262–3.594)	0.965	0.215 (0.035–1.316)	0.096
Comorbidity burden, per additional comorbidity	1.370 (0.937–2.004)	0.104	1.324 (0.857–2.046)	0.206	1.638 (0.913–2.937)	0.098

Abbreviations: aOR, adjusted odds ratio; CI, confidence interval; NIV, non-invasive ventilation; IMV, invasive mechanical ventilation; RSV, respiratory syncytial virus.

## Data Availability

The data presented in this study are available on request from the corresponding authors. The data are not publicly available due to intellectual property concerns.

## Abbreviations

The following abbreviations are used in this manuscript:

RSV	Respiratory Syncytial Virus
NIV	Non-invasive Mechanical Ventilation
IMV	Invasive Mechanical Ventilation
ICU	Intensive Care Unit
ARIs	Acute Respiratory Tract Infections
RT-PCR	Real-Time Polymerase Chain Reaction
CT	Computed Tomography
CRP	C-Reactive Protein
PCT	Procalcitonin
SD	Standard Deviation
IQR	Interquartile Range
